# A short review on minimally invasive implants

**DOI:** 10.6026/97320630019655

**Published:** 2023-05-31

**Authors:** Saathvika Ramani, R Vijayalakshmi, C. Burnice Nalina Kumari, Jaideep Mahendra, N Ambalavanan

**Affiliations:** 1 Department of Periodontology, Faculty of Dentistry, Meenakshi Academy of Higher Education and Research, Chennai 600 095, Tamil Nadu, India

**Keywords:** Minimally invasive implants, flapless implants, punch technique, direct drill technique, mini-incision technique

## Abstract

In the last 30 years, the use of dental implants to replace missing teeth has increased immensely. Brånemark pioneered the use of extensive surgical flaps to visualise the surgical field during implant surgery. Since then, several changes have been made to the flap design with aesthetic considerations now being incorporated. Such major innovations have contributed to the wide acceptance of flapless implant surgery. Therefore, it is of interest to describe the various techniques, requirements, advantages and disadvantages of minimally implant surgery.

## Background:

Periodontitis is one of the most common causes of tooth loss. In the last 30 years, the use of dental implants to replace missing teeth has increased immensely. [1] Brånemark pioneered the use of extensive surgical flaps to visualise the surgical field during implant surgery in the late 1970's. Several changes have been made to the flap design over the last three decades, with aesthetic considerations now being incorporated in the pivotal aesthetic zones of the dentition. In scenarios where bone quantity is limited, elevating a mucoperiosteal flap can help by enabling the surgeon to visually evaluate bone quantity and morphology at the site. However, flap elevation is always associated with some degree of morbidity and discomfort and the surgical wound must be sutured. Studies in the early 1970's found a link between flap elevation and gingival recession, as well as bone resorption around natural teeth. Furthermore, post-surgical tissue loss from flap elevation has been reported, implying that using flap surgery for implant placement may have a negative impact on implant aesthetic outcomes, particularly in the anterior maxilla. Over the last 30 years, flap designs for implant surgery have evolved, and more lately, the concept of implant placement without flap elevation and osseous exposure has been introduced. [2] Minimally invasive surgery or Flapless surgery involves accessing the bone by either (a) Soft tissue punch technique or (b) Direct drill technique or (c) Mini-incision technique.

## Punch technique:

A circumferential incision is placed on the gingiva at the middle of the implant site employing a surgical template. The cut is created with a soft tissue punch at low speed (100 rpm). The punch should be at least 1 mm wider than the implant to be placed. The incised gingival tissue is removed with a curette or mosquito hemostat.

## Direct drill technique:

The region of placement of implant is marked on the soft tissue employing a surgical template and the osteotomy site preparation is completed with conventional drills, drilling directly through the soft tissue within the marked area.

## Mini-incision technique:

A crestal mini-incision of roughly 5 mm is placed horizontal to the alveolar crest at the middle of the implant site. Local undermining of the gingiva is then performed. The quantity of undermining of the implant site must not exceed 5 mm. During this procedure, the soft tissue on each sides of the incision line is moved aside to accommodate the drills and implants. A brand new drill is employed to forestall damage to the adjacent soft tissue while drilling through a minute opening. The drill should have a cutting surface on its tip and rounded surfaces on all other sides to shield the soft tissue adjacent to the drill blades during contact with the rotating blades. Osteotomy preparation is then preceded consistent with the drill sequence. After fixture placement, a cover screw is connected and the incised gingiva is sutured to submerge the fixture. [3]

## Requirements of flapless technique:

The flapless technique could also be considered in conjunction with either single-stage placement or immediate loading. The principles that has to be observed during the procedure are: [4,5, 6,7]

[1] Keratinized, attached, and non-mobile tissue of at least 5 mm must be present, because the flapless procedure requires the definite removal of some amount of the tissue. This is crucial to provide the epithelial and connective tissue components needed for soft tissue integration and the development of circumferential biological width, without sacrificing the underlying peri-implant supporting bone.

[2] Bone width of at least 4.5 mm must be available without undercuts of greater than 15°. Since visibility is restricted when using the flapless technique, it is difficult to confirm that the implant is positioned in the center of the crestal bone. Greater ridge width offers the practitioner an additional margin of safety.

## Advantages of flapless surgery:

[1] Reduction of complications at the patient level, i.e. swelling and pain

[2] Reduction of intra-operative bleeding

[3] Reduction of surgical time and the need for suturing

[4] Preservation of soft and hard tissues

[5] Maintenance of blood supply. [2]

## Shortcomings of flapless surgery:

[1] The inability of the surgeon to visualize anatomical landmarks and vital structures,

[2] The potential for thermal trauma to the bone due to limited external irrigation during preparation of the osteotomy with guided surgery,

[3] Inability to ideally visualize the vertical endpoint of the implant placement (too shallow/too deep),

[4] Decreased access to the bony contours for alveoloplasty

[5] Inability to manipulate the circumferential soft tissues to ensure the ideal dimensions of keratinized mucosa around the implant.[2]

Ozan *et al.* (2007) [8] in his study using the punch method concluded that implants placed using flapless technique with CT-guided surgical stents could be possible and yield a high success rate. However, Sennerby *et al.* (2008) in his study concluded that for implants placed with the flapless technique using the punch method, the failure rate was significantly higher, and marginal bone resorption was slightly lower. [9]

De Bruyn *et al.* (2011) in his study concluded that single implants installed with flapless surgery using the direct drill technique, showed equal clinical success as those installed with conventional flap surgery. [10] Sunitha and Sapthagiri (2013) in their study using drill preparation technique, after 24 months follow-up, concluded that flapless implant surgery resulted in lesser loss of interproximal bone and also resulted in better papillary fill when compared with the flap technique. [11]

Jeong *et al.* (2009) in his study using the mini-incision technique, concluded that the mini-incision implant technique can be used at sites where implants need to be protected below the soft tissue during the early phase of healing, particularly for patients with poor bone quality and/or primary implant stability. [3]

Because of the limited access in these techniques, soft tissue contact during the flapless implant procedure may contaminate the implant surface. Some authors have argued that it is critical to prevent bacteria and biologic molecules (such as saliva and foreign bodies) from contaminating the implant surface during surgical insertion of implants. Nevertheless, after examining the differences in bony contact between biologically contaminated implants and standard control implants, Ivanoff *et al.* reported that pre-operative soft tissue contamination of titanium implants did not prevent osseointegration. [12] The above data support the utilization of these flapless implant techniques.

To assess the effect of flapless surgery on soft tissue, Oh *et al.* (2006) conducted a study comparing immediate loading and delayed (after 4 months) loading protocols. A flapless approach was chosen for both groups. Probing depths, modified bleeding index, modified plaque index as well as the width of keratinized gingiva were assessed. He concluded that there was no significant difference between the groups at each interval and over 6 months.[13]

Sclar *et al.* [4] reviewed the recommendations for flapless implant surgery, with special emphasis on requirements for establishing or maintaining long-term health and stability of the peri-implant soft tissues. The author stressed on establishing an adequate zone of attached, keratinized soft tissue of thickness 2.5-3 mm. This greatly contributes to the preservation of a stable peri-implant soft tissue environment. [2]

In terms of long-term success, according to Albrektsson's success criteria [14], the average marginal bone loss during the first year of functional use of an implant should be <1.5 mm. One year after conventional flap implant surgery, marginal bone loss has been reported to range from 0.4 to 1.2 mm. According to a study by Seung-Mi Jeong *et al.* (2011), the average bone loss one year after flapless implant surgery was 0.3 mm; no implants failed to osseointegrate, and no implants had bone loss greater than 1.2 mm. The authors attributed this to the preservation of the periosteum, which may aid in the healing of the peri-implant tissue, the use of a tissue punch narrower than the implant itself, and effective and early plaque control after implant placement. [12]

According to a long-term study by Pisoni Luca *et al.* (2016), during a 36 month follow-up period, between flapless and a traditional surgery, there were no statistical differences in bone resorption patterns between the two groups. Bone resorption did not differ significantly among the two groups at any of the three times taken into account which was at the time of implant placement, implant loading and at 36 months follow-up.[15] In a systematic review by Lin *et al.* (2014), several peri-implant parameters, including Probing depth (PD), Gingival index (GI), Plaque index (PI), and modified Plaque index (mPI), were evaluated to investigate the effect of the flapless procedure on the peri-implant tissue health. Most studies did not show statistically significant differences in the above examined parameters. [16] In one study,[17] that showed significantly higher PI and GI in the flapless procedure at short term (3 to 9 months), those differences were no longer statistically significant at 15 months. Therefore, the results demonstrated that the flapless procedure could achieve long-term peri-implant soft tissue health, which was similar to the traditional flap approach. As a result, it is reasonable to state that there is insufficient evidence that the flapless procedure can preserve marginal bone; thus, may have no long-term aesthetic benefit and should not be highly recommended for cases aiming for aesthetic outcomes. [16]

## Conclusion:

Minimally invasive implant surgery aims to minimize trauma to both the bone and soft tissues, reducing surgery time and thereby, attain higher levels of patient satisfaction. The main disadvantage of flapless surgery is the lack of visibility during drilling and implant placement, which raises the risk of causing incorrect bone angulations or damaging neighbouring structures. Another significant disadvantage of the technique would be the inability to perform bone regeneration or soft tissue handling techniques. Therefore, flapless surgeries should be confined to carefully selected cases with proper clinical and radiological planning. The choice of flapless or flap surgery for implant placement remains entirely in the hands of the operating dentist, his/her skill level and the requirements of the individual patient.
